# Microbiota disparities in stool, oral swabs, and saliva between control and early-onset colorectal neoplasia groups: an exploratory analysis

**DOI:** 10.3389/frmbi.2026.1687978

**Published:** 2026-02-04

**Authors:** Ji Eun Na, Tae Oh Kim, Yong Eun Park

**Affiliations:** 1 Department of Internal Medicine, Bumin Hospital Haeundae, Busan, Republic of Korea; 2 Department of Internal Medicine, Inje University Haeundae Paik Hospital, Busan, Republic of Korea

**Keywords:** adenoma, biomarkers, early-age onset colorectal neoplasia, microbiota, serrated lesions

## Abstract

**Background/aim:**

The increasing incidence of early-age-onset colorectal neoplasia (EAO-CRN) in individuals under 50 years old poses a global health concern. This study aimed to investigate the variations in the microbiota in individuals with EAO-CRN compared with a control group, utilizing stool, oral swab, and saliva samples.

**Methods:**

Participants under 50 years of age provided stool, oral swab, and saliva samples. Colorectal neoplasia was classified into the serrated lesions and adenoma–carcinoma groups based on histology and compared with a control group without polyps. The alpha diversity and the taxonomic abundance differences were assessed using amplicon sequence variants obtained through 16S rRNA sequencing and matched taxonomy data.

**Results:**

A total of 45 participants were included: 14 in the control, 13 in the serrated lesions, and 18 in the adenoma–carcinoma groups. Microbial analysis revealed no significant differences in the alpha diversity among the groups. However, the stool samples from the serrated lesions group had higher levels of the families Erysipelotrichaceae and Lachnospiraceae compared with the control group. Analysis of the oral swabs indicated relatively elevated levels of the family Streptococcaceae in both the serrated lesions and adenoma–carcinoma groups. In the saliva samples, the serrated lesions and adenoma–carcinoma groups showed higher levels of the family Lactobacillaceae, with the serrated lesions group also exhibiting elevated levels of the family Bifidobacteriaceae.

**Conclusions:**

This study elucidates the microbiota changes associated with EAO-CRN, distinguishing between serrated lesions and adenoma–carcinoma groups using stool, oral swab, and saliva samples. These findings contribute to the understanding of the relationship between microbiota and colorectal neoplasia in the early-onset population.

## Introduction

Early-age-onset colorectal neoplasia (EAO-CRN) encompasses neoplastic polyps and carcinomas occurring in individuals younger than 50 years. The incidence of EAO-CRN has increased steadily over recent decades, with the colorectal cancer (CRC) rates in younger adults rising by up to approximately 2% per year, raising concerns about a growing global burden (Bailey et al., 2015; Kolb et al., 2021; Jeong et al., 2022; Siegel et al., 2023; Tang et al., 2023). The underlying causes appear multifactorial and include genetic susceptibility, lifestyle factors (such as diet, obesity, and physical inactivity), inflammatory bowel disease, and environmental exposures (Patel et al., 2022; Adigun et al., 2023).

Although screening colonoscopy in individuals aged 50 years and older has reduced CRC incidence and mortality (Zauber et al., 2012; Nishihara et al., 2013; Bibbins-Domingo et al., 2016; Zhang et al., 2020; Shaukat et al., 2021; Bretthauer et al., 2022), both the incidence and the mortality of early-onset CRC (EO-CRC) continue to rise among those under 50 years (Araghi et al., 2019; Sinicrope, 2022). Compared with late-onset CRC, EO-CRC more frequently presents with left-sided or rectal tumors, advanced-stage disease, and unfavorable histologic features, suggesting distinct carcinogenic pathways and associated microbial alterations (Chang et al., 2012; Chang et al., 2012; Lieu et al., 2019; Serebriiskii et al., 2019; Willauer et al., 2019; Collaborative, 2021; Dharwadkar et al., 2021; Yang et al., 2021; Saraiva et al., 2023). In addition, the lack of a screening program and the diagnostic delays due to patient- or clinician-related factors contribute to the challenges of managing EO-CRC (Nishihara et al., 2013; Castelo et al., 2022). In response, recent guidelines have lowered the recommended age for CRC screening to 45 years (Meester et al., 2018; Peterse et al., 2018; Wolf et al., 2018; Davidson et al., 2021). Consequently, the increased detection of neoplastic polyps, which are recognized precursors of CRC, in younger individuals has heightened interest in the pathogenesis and management of EAO-CRN (Rundle et al., 2008; Lee et al., 2016; Yang et al., 2021; Jeong et al., 2022; Ma et al., 2023; Penz et al., 2023; Vithayathil et al., 2023).

A large cross-cohort meta-analysis demonstrated that CRC-associated microbial patterns are reproducible across cohorts and geographies, strengthening the rationale for microbiota profiling as a diagnostic adjunct (Wirbel et al., 2019). Within these signatures, specific taxa such as *Fusobacterium nucleatum* have shown diagnostic associations with CRC.36 (Thomas et al., 2019; Obón-Santacana et al., 2022; Yu et al., 2022; Pandey et al., 2023), In addition, multiple studies have linked gut microbiota dysbiosis with neoplastic polyps, indicating that microbial alterations may emerge early along colorectal tumorigenesis (Peters et al., 2016; Hale et al., 2017; Mangifesta et al., 2018; Rezasoltani et al., 2018). Recent studies also suggest that oral microbiota profiling may provide a noninvasive approach for early colorectal neoplasia (CRN) detection (Flemer et al., 2018; Zhang et al., 2023). However, research specifically focusing on the microbial changes in individuals with EAO-CRN remains limited (Yang et al., 2021).

Neoplastic polyps are typically classified into serrated lesions and adenomatous polyps based on their histology and distinct characteristics (De Palma et al., 2019; Nguyen et al., 2020). Accordingly, this study examined the microbiota changes in individuals with EAO-CRN compared with a control group using stool, oral swab, and saliva samples, with stratification by histologic subtype.

## Methods

### Studied individual selection, sampling, and categorization

This is a single-center cross-sectional study targeting individuals aged from 20 to under 50 years who underwent an index colonoscopy at Haeundae Paik Hospital from 2020 to 2022. The study population included adult patients in this age group who visited the gastroenterology outpatient clinic for screening purposes. The exclusion criteria were as follows: 1) a history of inflammatory bowel disease, intestinal tuberculosis, Behçet’s disease, infectious enterocolitis, or other functional gastrointestinal disorders (e.g., irritable bowel syndrome, constipation, or diarrhea); 2) use of antibiotics or probiotics in the past month; 3) a first-degree family history of CRC; 4) known genetic factors such as polyposis syndrome; and 5) being in a vulnerable state, including pregnancy or psychiatric disorders. Participation required voluntary written consent after a comprehensive explanation of the research, with only those who provided consent included in the study. The study protocol was approved by the Institutional Review Board of Inje University Haeundae Paik Hospital (file no. 2019-11-027-008).

Stool, oral swab, and saliva samples were collected prior to colonoscopy. Stool samples were obtained using a dedicated sterile container. Oral swabs were gently collected from the inner cheeks using a sterile swab and placed into sterile collection tubes. Saliva was collected as a clear liquid with a volume of at least 5 ml, at least 1 h after food intake and tooth brushing, into sterile saliva collection tubes. All specimens were transported to the analysis laboratory at Macrogen Inc. under cold-chain conditions and stored at −80°C until DNA extraction.

The study participants were categorized according to their colonoscopy findings and histopathologic diagnoses as follows (Gupta et al., 2020; Rutter et al., 2020; Kim et al., 2023): 1) control, no polyps detected; 2) serrated lesions, including hyperplastic polyps in the proximal colon (excluding distal hyperplastic polyps), sessile serrated lesions, and traditional serrated adenomas; and 3) adenomatous polyps and carcinoma. Accordingly, the final classification comprised the control group, the serrated lesions group, and the adenoma–carcinoma group.

### Description of the studied individuals

A total of 50 individuals participated in the study, with one excluded due to a juvenile polyp found in the pathology results. Consequently, specimens from 49 individuals were enrolled for stool, oral swab, and saliva analysis (**Table 1**). The median age was 42 years, and sex distribution was approximately balanced.

**Table 1:** Baseline characteristics of the enrolled individuals.

Total no. of participants	49
Age (years)	42 (39–45)
Men/women	25/24 (51.0%/49.0%)
Group categories	
Control (no polyp)	14 (28.6%)
Hyperplastic polyps, distal and diminutive	4 (8.2%)
Serrated lesions	13 (26.5%)
Adenoma–carcinoma	18 (36.7%)

Categorical variables are presented as numbers and percentages, whereas continuous variables are represented as median and interquartile range.

Among the 49 participants, 14 individuals (28.6%) were the controls without polyps. The remaining participants comprised 4 (8.2%) with distal diminutive hyperplastic polyps, 13 (26.5%) with serrated lesions, and 18 (36.7%) in the adenoma–carcinoma group, including one case of carcinoma. As distal diminutive hyperplastic polyps were not a focus of the present study, these individuals were excluded from the final comparative analyses, leaving 45 participants for the primary analysis.

### Library construction and sequencing

The sequencing libraries were prepared following the Illumina 16S Metagenomic Sequencing Library protocols to amplify the V3 and V4 regions. A 5-ng input of genomic deoxyribonucleic acid (gDNA) underwent polymerase chain reaction (PCR) amplification using 5× reaction buffer, 1 mM of dNTP mix, 500 nM each of the universal forward/reverse PCR primer, and Herculase II fusion DNA polymerase (Agilent Technologies, Santa Clara, CA, USA). The cycle conditions for the first PCR included a 3-min heat activation at 95°C, followed by 25 cycles of 30 s at 95 °C, 30 s at 55°C, and 30 s at 72°C, with a final extension at 72°C for 5 min. The universal primer pair with Illumina adapter overhang sequences used for the first amplification were as follows:

16S amplicon PCR forward primer: 5' TCGTCGGCAGCGTCAGATGTGTATAAGAGACAGCCTACGGGNGGCWGCAG.

16S amplicon PCR reverse primer: 5' TCTCGTGGGCTCGGAGATGTGTATAAGAGACAGGACTACHVGGGTATCTAATCC.

The first PCR product was purified with AMPure beads (Agencourt Bioscience, Beverly, MA, USA). Following purification, 10 μl of the first PCR product underwent a second PCR amplification for final library construction, including the index, using NexteraXT Indexed Primer. The cycle conditions for the second PCR were the same as those for the first PCR, except for 10 cycles. The PCR product was again purified with AMPure beads. The final purified product was then quantified using quantitative PCR (qPCR) according to the qPCR Quantification Protocol Guide (KAPA Library Quantification kits for Illumina Sequencing platforms) and qualified using the TapeStation D1000 ScreenTape (Agilent Technologies, Waldbronn, Germany). Sequencing was performed using the MiSeq™ platform (Illumina, San Diego, CA, USA).

### Amplicon sequence variant analysis

After the completion of Illumina MiSeq sequencing, the raw data were sorted by sample using the index sequences, and paired-end FASTQ files were generated for each sample. Preprocessing was carried out using the Cutadapt (version 3.2) (Martin, 2011) program to remove the sequencing adapter sequences and the target gene region F/R primer sequences. Subsequently, the forward sequence (Read1) and the reverse sequence (Read2) were trimmed to 250 and 200 bp, respectively.

For error correction in the amplicon sequencing process, the DADA2 version 1.18.0 package in the R version 4.0.3 program was utilized (Callahan et al., 2016). Sequences with expected errors greater than or equal to 2 were excluded from consideration in the paired-end reads. After preprocessing, an error model was established for each batch to remove sample-specific noise. The error-corrected paired-end sequences were assembled into a single sequence, and the DADA2 Consensus method was employed to remove chimera sequences and form amplicon sequence variants (ASVs). Among the generated ASVs, those shorter than 350 bp were excluded using R version 4.0.3.

For microbial community comparison analysis, subsampling was applied for normalization of the alpha diversity and other diversity-based comparisons using the QIIME 1.9 program (Caporaso et al., 2010). This involved selecting the minimum read count among all samples as the threshold for subsampling across the entire set of samples. These procedures are also available in QIIME 2 using the same underlying scripts. For computational efficiency, several pipeline components (including stepwise utilities such as DADA2 and QIIME 2 internal tools) were installed and executed directly on the analysis server. This involved selecting the minimum read count among all samples as the threshold for subsampling across the entire set of samples.

Each ASV sequence was subjected to BLAST+ version 2.9.0 (Camacho et al., 2009) against the reference database (DB) (NCBI 16S Microbial DB). Taxonomy information for the organism with the highest similarity to the subject was assigned. However, if the query coverage of the best hit in the database was less than 85% or if the identity of the matched region was less than 85%, taxonomy information was not assigned. The MAFFT version 7.475 was employed for multiple alignments of the ASV sequences. Subsequently, a phylogenetic tree was generated using the FastTreeMP version 2.1.10 program (Price et al., 2010). Using the taxonomy information obtained from the ASVs, various microbial community comparison analyses were conducted using QIIME.

### Statistical analysis

Alpha diversity metrics, including ASVs and the Shannon, Gini–Simpson, and Chao1 indices, were calculated to assess the microbial diversity within samples. For between-group comparisons of the taxonomic composition, linear discriminant analysis effect size (LEfSe) was performed (Segata et al., 2011). Briefly, taxa showing differential abundance between groups were first identified using the Kruskal–Wallis test, and linear discriminant analysis (LDA) was then used to estimate the effect size of each differentially abundant taxon. Taxa with an LDA score >2.0 and *p* < 0.05 were considered significant. Visualizations (histograms and box plots) were generated using R (version 3.6.2).

## Results

### Alpha diversity of the microbiota

The alpha diversity did not reveal significant differences in the stool, oral swab, and saliva samples when comparing the control group with the serrated lesions group or the adenoma–carcinoma group.

### Taxon differences in the stool samples

At higher taxonomic levels, the LEfSe showed that the control group was enriched in the Bacteroidota lineage, including the phylum Bacteroidota, the class Bacteroidia, and the order Bacteroidales (all LDA = 4.8, *p* = 0.04) (**Table 2**). At the family level, the control group exhibited higher abundance of Barnesiellaceae (LDA = 3.3, *p* = 0.04) and Feifaniaceae (LDA = 3.2, *p* = 0.01), whereas the serrated lesions group was characterized by higher abundance of Erysipelotrichaceae (LDA = 3.8, *p* = 0.02) and Lachnospiraceae (LDA = 4.7, *p* = 0.01).

**Table 2:** Taxonomic differences in the stool microbiota between the control, serrated lesions, and adenoma–carcinoma groups at the phylum to the family level.

Stool	Control group	LDA score (log10)	*p*	Serrated lesions group	LDA score (log10)	*p*
Phylum	Bacteroidota	4.8	0.04			
Class	Bacteroidia	4.8	0.04			
Order	Bacteroidales	4.8	0.04			
Family	Barnesiellaceae	3.3	0.04	Erysipelotrichaceae	3.8	0.02
	Feifaniaceae	3.2	0.01	Lachnospiraceae	4.7	0.01

	Control group	LDA score (log10)	*p*	Adenoma–carcinoma group	LDA score (log10)	*p*
Family	Atopobiaceae	2.7	0.01			
	Vallitaleaceae	2.7	0.02			

In the comparisons between the control and adenoma–carcinoma groups, the control group was characterized by a higher abundance of Atopobiaceae (LDA = 2.7, *p* = 0.01) and Vallitaleaceae (LDA = 2.7, *p* = 0.02).

**Figure 1:**
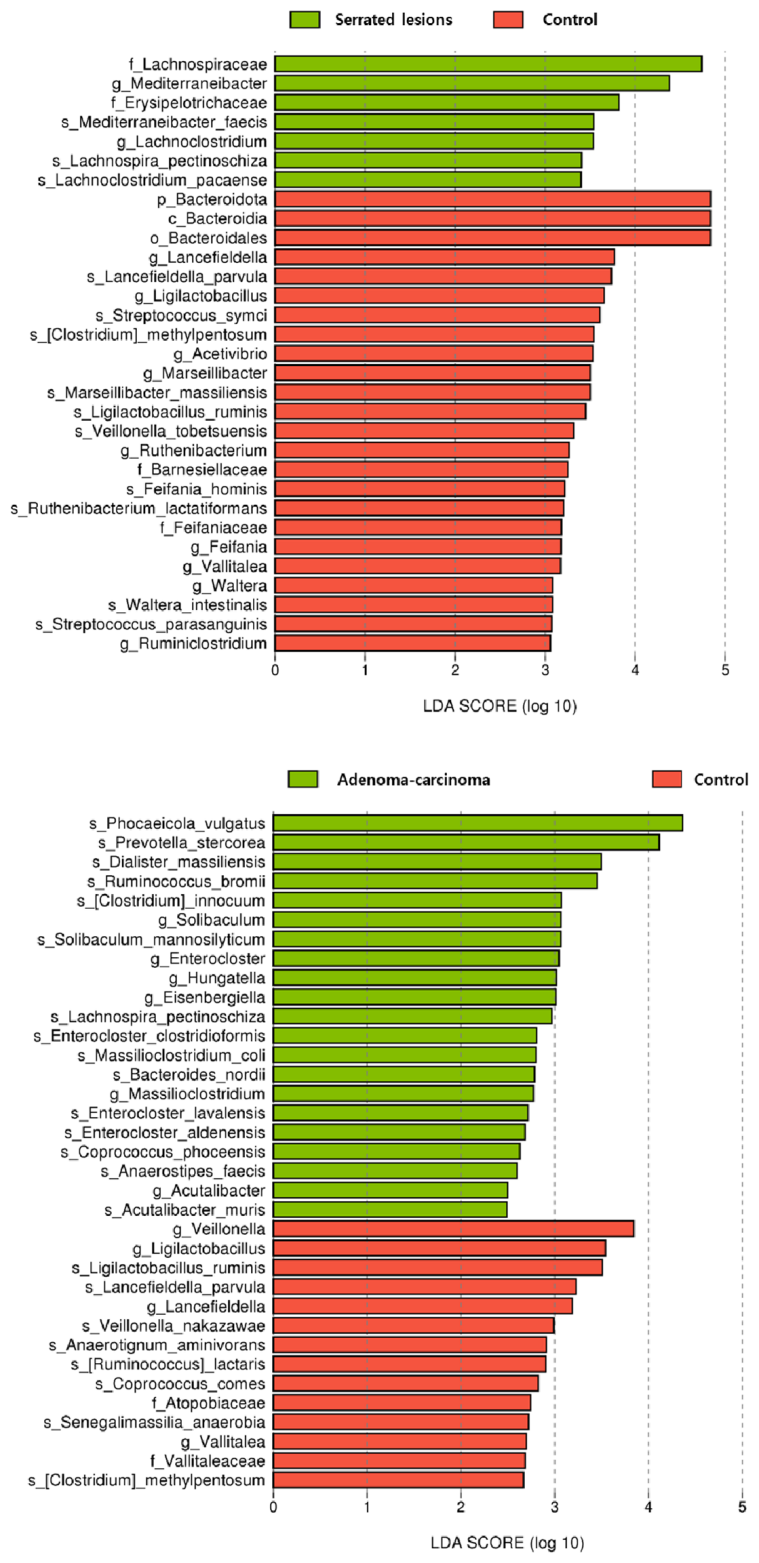
Taxonomic differences in the stool samples.

At the genus level, the control group demonstrated a higher abundance of several genera, including *Ligilactobacillus*, *Lancefieldella*, and *Marseillibacter* (all *p* < 0.05) (**Figure 1** and **Supplementary Table S1**). In contrast, the serrated lesions group exhibited higher relative abundance of *Mediterraneibacter* (LDA = 4.4, *p* = 0.01) and *Lachnoclostridium* (LDA = 3.5, *p* = 0.02). The species-level signals were directionally consistent with these genus-level patterns, with taxa such as *Mediterraneibacter faecis* and *Lachnoclostridium pacaense* enriched in the serrated lesions group.

In the comparisons between the control and adenoma–carcinoma groups, the control group was enriched in *Ligilactobacillus*, *Lancefieldella*, *Veillonella*, and *Vallitalea* (all *p* < 0.05). Conversely, the adenoma–carcinoma group showed an increased abundance of the genera associated with dysbiosis, including *Eisenbergiella*, *Enterocloster*, and *Hungatella* (all *p* < 0.05). Detailed taxonomic results are provided in **Supplementary Table S1**.

### Taxon differences in the oral swabs

**Figure 2:**
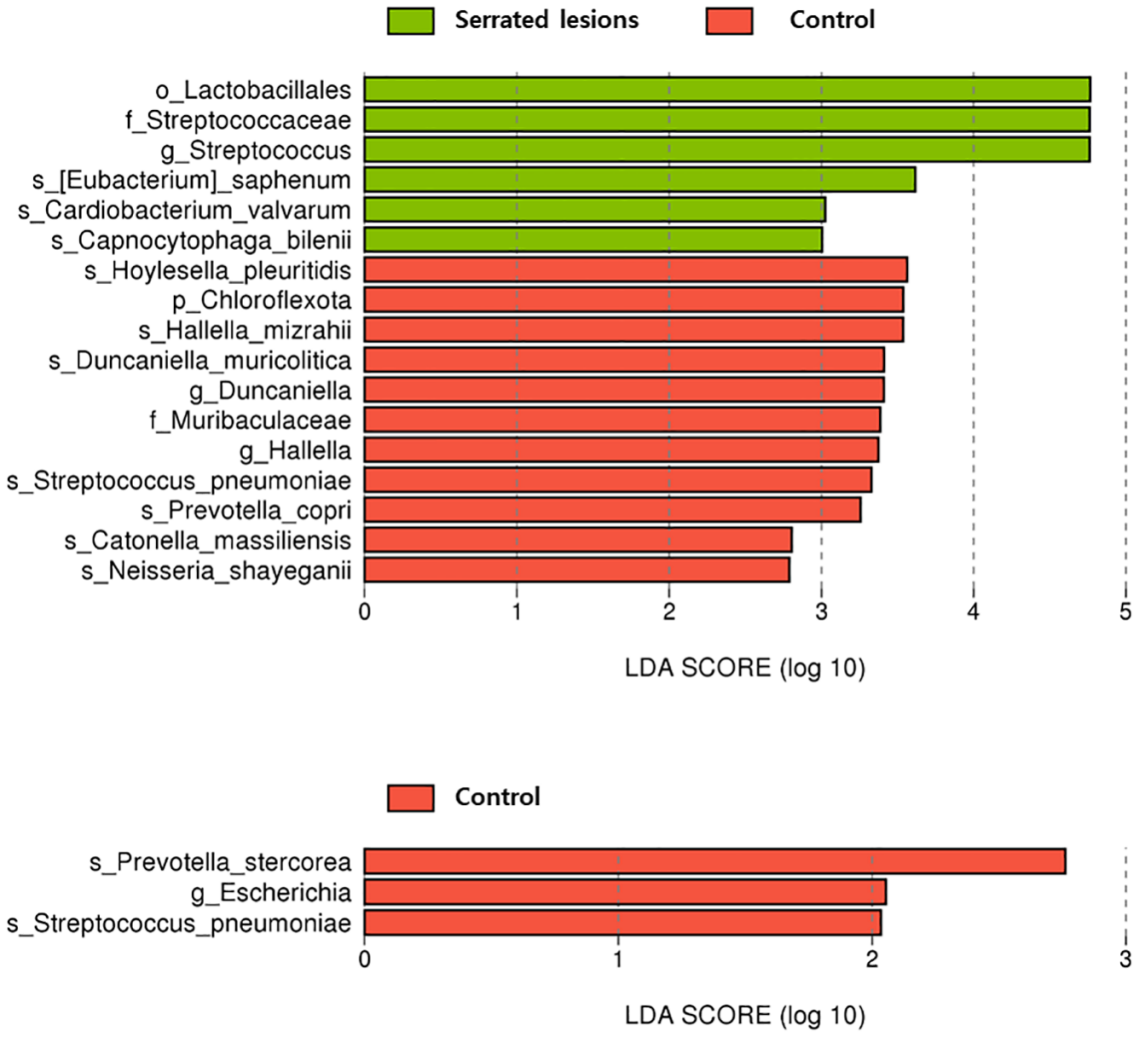
Taxonomic differences in the oral swab samples.

In the oral samples, the control group showed enrichment of the phylum Chloroflexota (LDA = 3.5, *p* = 0.04) and the family Muribaculaceae (LDA = 3.4, *p* = 0.02). In contrast, the serrated lesions group was characterized by enrichment of the order Lactobacillales (LDA = 4.8, *p* = 0.03) and the family Streptococcaceae (LDA = 4.8, *p* = 0.05) (**Table 3** and **Figure 2**).

**Table 3:** Taxonomic differences in the oral swab and saliva microbiota between the control, serrated lesions, and adenoma–carcinoma groups at the phylum to the family level.

Oral swabs	Control group	LDA score (log10)	*p*	Serrated lesions group	LDA score (log10)	*p*
Phylum	Chloroflexota	3.5	0.04			
Order				Lactobacillales	4.8	0.03
Family	Muribaculaceae	3.4	0.02	Streptococcaceae	4.8	0.05

Saliva	Control group	LDA score (log10)	*p*	Serrated lesions group	LDA score (log10)	P
Order				Bifidobacteriales	2.5	0.01
Family	Weeksellaceae	3.2	0.04	Bifidobacteriaceae	2.5	0.01
				Lactobacillaceae	2.3	0.01

At the genus level, the control group was enriched in *Duncaniella* (LDA = 3.4, *p* = 0.04) and *Hallella* (LDA = 3.4, *p* = 0.03), whereas the serrated lesions group showed enrichment of *Streptococcus* (LDA = 4.8, *p* = 0.04) (**Supplementary Table S2**).

In the comparisons between the control and adenoma–carcinoma groups, no taxa reached statistical significance at the family level or higher. At the genus level, only *Escherichia* was identified as a discriminant taxon in the control group (LDA = 2.1, *p* = 0.04). The species-level results are provided in **Supplementary Table S2**.

### Taxon differences in the saliva samples

**Figure 3:**
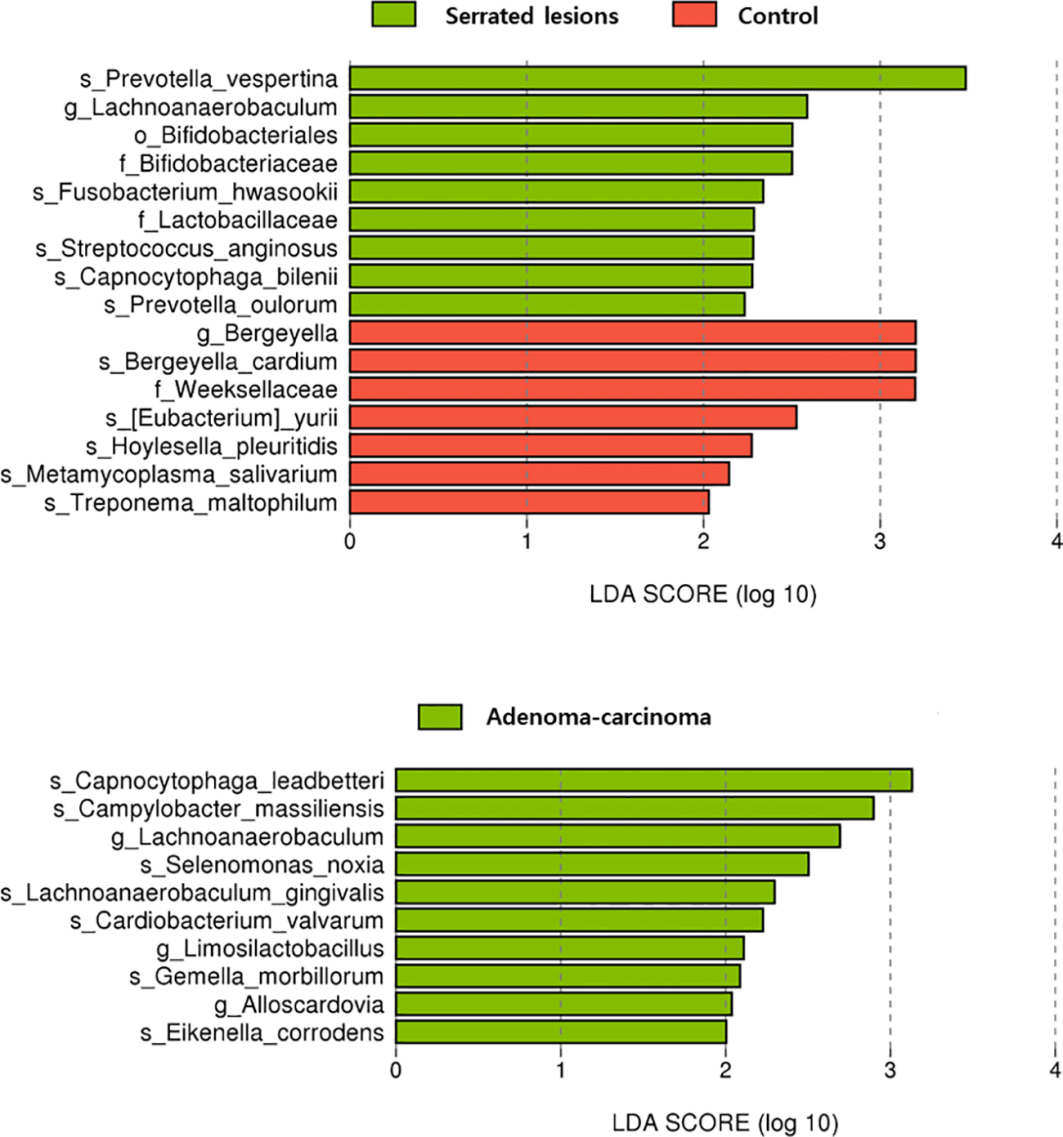
Taxonomic differences in the saliva samples.

At higher taxonomic levels, the control group showed enrichment of the family Weeksellaceae (LDA = 3.2, *p* = 0.04). In contrast, the serrated lesions group was characterized by enrichment of the order Bifidobacteriales (LDA = 2.5, *p* = 0.01) and the families Bifidobacteriaceae (LDA = 2.5, *p* = 0.01) and Lactobacillaceae (LDA = 2.3, *p* = 0.01) (**Table 3** and **Figure 3**).

In contrast, no taxa reached statistical significance at the family level or higher in the comparison between the control and the adenoma–carcinoma group.

At the genus level, the control group was enriched in *Bergeyella* (LDA = 3.2, *p* = 0.04), whereas the serrated lesions group was characterized by enrichment of *Lachnoanaerobaculum* (LDA = 2.6, *p* = 0.03). In the control *versus* adenoma–carcinoma group comparison, discriminant genera were observed only in the adenoma–carcinoma group, including *Alloscardovia* (LDA = 2.0, *p* = 0.04), *Lachnoanaerobaculum* (LDA = 2.7, *p* = 0.01), and *Limosilactobacillus* (LDA = 2.1, *p* = 0.04). The species-level findings are presented in **Supplementary Table S3**.

## Discussion

We analyzed microbiota changes in individuals with EAO-CRN compared with a control group using stool, oral swab, and saliva samples. The alpha diversity metrics did not reveal significant differences between the control group and either the serrated lesions or the adenoma-carcinoma group. However, differences in the taxonomic composition were observed, particularly when comparing the control group with the pathologically classified groups. In the stool samples, the serrated lesions group exhibited higher relative abundance of the families Erysipelotrichaceae and Lachnospiraceae compared with the control group. In the oral swab samples, the serrated lesions group showed enrichment of the family Streptococcaceae. In the saliva samples, higher relative abundance of Bifidobacteriaceae and Lactobacillaceae was observed in the serrated lesions group. In contrast, no taxa at or above the family level showed statistically significant differences between the control and the adenoma–carcinoma group in either the oral swab or the saliva samples.

This study has several strengths. Firstly, it is among the first to investigate microbiota alterations in EAO-CRN using stool, oral swab, and saliva samples. Secondly, it demonstrates originality by stratifying EAO-CRN into serrated lesions and adenoma–carcinoma groups based on histopathology, thereby addressing the hypothesis that microbial alterations may differ according to distinct carcinogenic pathways (Nguyen et al., 2020; DeDecker et al., 2021). Thirdly, unlike the majority of previous studies that primarily compared the microbiomes of patients with confirmed CRC to those of healthy controls (Pandey et al., 2023; Zwezerijnen-Jiwa et al., 2023), this study addresses a critical gap. This approach enables exploration of potential stepwise microbial shifts along the CRN continuum. Finally, given the increasing incidence of EAO-CRN and the current lack of reliable noninvasive biomarkers, our findings highlight the potential clinical utility of microbiome profiling for risk stratification and disease characterization. Collectively, this study provides a foundation for future investigations into microbiome-associated mechanisms involved in EAO-CRN development.

Research on microbial changes specifically related to EAO-CRN is limited, making direct comparisons with other studies challenging. However, when compared with studies investigating microbial differences between colon polyps and healthy controls (Shen et al., 2010; Peters et al., 2016; Hale et al., 2017; Mangifesta et al., 2018; Rezasoltani et al., 2018; Zhang et al., 2023), several overlapping patterns can be observed. For instance, in the stool samples, the control group showed higher relative abundance of the phylum Bacteroidota and the genus *Veillonella* (Shen et al., 2010; Hale et al., 2017). In the oral swab and saliva samples, the CRN group exhibited relatively higher levels of the genus *Streptococcus* (Chen et al., 2013; Peters et al., 2016; Mangifesta et al., 2018), aligning with previous studies that have identified *Streptococcus* as an organism increasingly associated with colorectal carcinogenesis (Pandey et al., 2023). Notably, these taxa also demonstrated high LDA scores in our analysis, supporting their potential utility as discriminatory microbial markers. In addition, the species *Gemella morbillorum*, previously reported to be enriched in CRC compared with controls in stool-based studies, was similarly observed in the adenoma–carcinoma group in our saliva samples (Thomas et al., 2019; Zhang et al., 2021; Li et al., 2022). These findings suggest that future research should focus on more sequential and detailed studies using a variety of sample types beyond stool. Notably, while previous studies have observed higher levels of the family Lachnospiraceae in healthy groups or reported negative correlations with disease groups (Zackular et al., 2014; Peters et al., 2016; Mangifesta et al., 2018; Pandey et al., 2023), our study indentified increased levels of Lachnospiraceae in the serrated lesions group compared with the controls. This discrepancy suggests that the microbial signatures may differ according to polyp subtype, highlighting the biological heterogeneity of CRN and the need for stratified analyses in future studies.

This study has several limitations. Firstly, the sample size was relatively small, which may limit the statistical power to detect subtle microbial differences associated with EAO-CRN. Although we identified taxa that differed significantly between the CRN group (including both the serrated lesions and adenoma–carcinoma groups) and the control group, the limited sample size may have constrained our ability to identify robust or generalizable microbial signatures predictive of EAO-CRN. However, as demonstrated in prior CRC research, reproducible microbial patterns often emerge through cumulative evidence across independent studies, suggesting that future larger-scale investigations may further clarify these associations. Secondly, although we accounted for factors such as antibiotic and probiotic use before sample collection, other potential confounding factors, including diet, obesity, smoking, and alcohol, were not fully controlled, which may have influenced the observed microbial profiles. Thirdly, while we analyzed stool, oral swab, and saliva samples, the microbial composition may differ between luminal contents and mucosa-associated communities, which were not directly assessed in this study (Chen et al., 2012). Finally, this study was conducted exclusively in a Korean population; therefore, the generalizability of the findings to other ethnic or geographic populations may be limited. Future studies involving more diverse cohorts will be necessary to validate and extend these observations (Elkholy et al., 2023).

This study demonstrated distinct microbial differences between individuals with EAO-CRN and control subjects using stool, oral swab, and saliva samples. By accounting for differences in the carcinogenic pathways and stratifying EAO-CRN into serrated lesions and adenoma–carcinoma groups, our findings suggest that microbial alterations may vary according to the underlying pathway of tumorigenesis. These observations underscore the potential clinical relevance of microbiome-based profiling, particularly for the development of noninvasive biomarkers aimed at the early detection and risk stratification of EAO-CRN.

## Data Availability

The minimal dataset underlying the findings of our study cannot be made publicly available due to ethical and patient privacy restrictions. The data are available from the corresponding author upon reasonable request and with appropriate approvals.
