# Supplementary material for: Microbiota disparities in stool, oral swabs, and saliva between control and early-onset colorectal neoplasia groups: an exploratory analysis
Source: Front Microbiomes. 2026 Feb 4;5:1687978. doi: 10.3389/frmbi.2026.1687978 (PMC12993686; doi:10.3389/frmbi.2026.1687978)
Supplement: Supplementary file 1 [file Table1.docx]

Supplementary Table 1. Genus and species level taxonomic differences in stool microbiota among control, serrated lesion, and adenoma–carcinoma groups

| Stool | Control | LDA score (log10) | P | Serrated lesions | LDA score (log10) | P |
| --- | --- | --- | --- | --- | --- | --- |
| Geuns | Acetivibrio | 3.5 | 0.04 | Lachnoclostridium | 3.5 | 0.02 |
|  | Feifania | 3.2 | 0.01 | Mediterraneibacter | 4.4 | 0.01 |
|  | Lancefieldella | 3.8 | 0.04 |  |  |  |
|  | Ligilactobacillus | 3.7 | 0.02 |  |  |  |
|  | Marseillibacter | 3.5 | 0.02 |  |  |  |
|  | Ruminiclostridium | 3.1 | 0.04 |  |  |  |
|  | Ruthenibacterium | 3.3 | 0.04 |  |  |  |
|  | Vallitalea | 3.2 | 0.04 |  |  |  |
|  | *Waltera* | 3.1 | 0.02 |  |  |  |
| Spieces | Clostridium methylpentosum | 3.5 | 0.03 | Mediterraneibacter faecis | 3.5 | 0.03 |
|  | Feifania hominis | 3.2 | 0.01 | Lachnoclostridium pacaense | 3.4 | 0.04 |
|  | Lancefieldella parvula | 3.7 | 0.04 | Lachnospira pectinoschiza | 3.4 | 0.01 |
|  | Ligilactobacillus ruminis | 3.4 | 0.02 |  |  |  |
|  | Marseillibacter massiliensis | 3.5 | 0.02 |  |  |  |
|  | Ruthenibacterium lactatiformans | 3.2 | 0.04 |  |  |  |
|  | Streptococcus parasanguinis | 3.1 | 0.03 |  |  |  |
|  | Streptococcus symci | 3.6 | 0.04 |  |  |  |
|  | Veillonella tobetsuensis | 3.3 | 0.02 |  |  |  |
|  | Waltera intestinalis | 3.1 | 0.02 |  |  |  |
|  | Control | LDA score (log10) | P | Adenoma-carcinoma | LDA score (log10) | P |
| Genus | Lancefieldella | 3.2 | 0.02 | Acutalibacter | 2.5 | 0.02 |
|  | Ligilactobacillus | 3.5 | 0.02 | Eisenbergiella | 3.0 | 0.03 |
|  | Vallitalea | 2.7 | 0.02 | Enterocloster | 3.0 | 0.01 |
|  | Veillonella | 3.8 | 0.03 | Hungatella | 3.0 | 0.01 |
|  |  |  |  | Massilioclostridium | 2.8 | 0.04 |
|  |  |  |  | Solibaculum | 3.1 | 0.01 |
| Spieces | Anaerotignum aminivorans | 2.9 | 0.04 | Acutalibacter muris | 2.5 | 0.02 |
|  | Clostridium methylpentosum | 2.7 | 0.04 | Anaerostipes faecis | 2.6 | 0.04 |
|  | Coprococcus comes | 2.8 | 0.04 | Bacteroides nordii | 2.8 | 0.04 |
|  | Lancefieldella parvula | 3.2 | 0.02 | Clostridium innocuum | 3.1 | 0.04 |
|  | Ligilactobacillus ruminis | 3.5 | 0.01 | Coprococcus phoceensis | 2.6 | 0.04 |
|  | Ruminococcus lactaris | 2.9 | 0.01 | Dialister massiliensis | 3.5 | 0.02 |
|  | Senegalimassilia anaerobia | 2.7 | 0.02 | Enterocloster aldenensis | 2.7 | 0.04 |
|  | Veillonella nakazawae | 3.0 | 0.03 | Enterocloster clostridioformis | 2.8 | 0.01 |
|  |  |  |  | Enterocloster lavalensis | 2.7 | 0.01 |
|  |  |  |  | Lachnospira pectinoschiza | 3.0 | 0.02 |
|  |  |  |  | Massilioclostridium coli | 2.8 | 0.04 |
|  |  |  |  | Phocaeicola vulgatus | 4.4 | 0.02 |
|  |  |  |  | Prevotella stercorea | 4.1 | 0.04 |
|  |  |  |  | Ruminococcus bromii | 3.5 | 0.04 |
|  |  |  |  | Solibaculum mannosilyticum | 3.1 | 0.01 |

Supplementary Table 2. Genus and species level taxonomic differences in oral swab microbiota among control, serrated lesion, and adenoma–carcinoma groups

| Oral swab | Control | LDA score (log10) | P | Serrated lesions | LDA score (log10) | P |
| --- | --- | --- | --- | --- | --- | --- |
| Geuns | Duncaniella | 3.4 | 0.04 | Streptococcus | 4.8 | 0.04 |
|  | Hallella | 3.4 | 0.03 |  |  |  |
| Spieces | Catonella massiliensis | 2.7 | 0.04 | Capnocytophaga bilenii | 3.0 | 0.04 |
|  | Duncaniella muricolitica | 3.4 | 0.04 | Cardiobacterium valvarum | 3.0 | 0.02 |
|  | Hallella mizrahii | 3.5 | 0.01 | Eubacterium saphenum | 3.6 | 0.03 |
|  | Hoylesella pleuritidis | 3.6 | 0.01 |  |  |  |
|  | Neisseria shayeganii | 2.8 | 0.04 |  |  |  |
|  | Prevotella copri | 3.3 | 0.04 |  |  |  |
|  | Streptococcus pneumoniae | 3.3 | 0.04 |  |  |  |
|  | Control | LDA score (log10) | P | Adenoma-carcinoma | LDA score (log10) | P |
| Genus | Escherichia | 2.1 | 0.04 |  |  |  |
| Spieces | Prevotella stercorea | 2.8 | 0.04 |  |  |  |
|  | Streptococcus pneumoniae | 2.0 | 0.02 |  |  |  |

Supplementary Table 3. Genus and species level taxonomic differences in saliva microbiota among control, serrated lesion, and adenoma–carcinoma groups

| Saliva | Control | LDA score (log10) | P | Serrated lesions | LDA score (log10) | P |
| --- | --- | --- | --- | --- | --- | --- |
| Geuns | Bergeyella | 3.2 | 0.04 | Lachnoanaerobaculum | 2.6 | 0.03 |
| Spieces | Bergeyella cardium | 3.2 | 0.04 | Capnocytophaga bilenii | 2.3 | 0.04 |
|  | Eubacterium yurii | 2.5 | 0.04 | Fusobacterium hwasookii | 2.3 | 0.04 |
|  | Hoylesella pleuritidis | 2.3 | 0.02 | Prevotella oulorum | 2.2 | 0.04 |
|  | Metamycoplasma salivarium | 2.1 | 0.02 | Prevotella vespertina | 3.5 | 0.03 |
|  | Treponema maltophilum | 2.0 | 0.04 | Streptococcus anginosus | 2.3 | 0.02 |
|  | Control | LDA score (log10) | P | Adenoma-carcinoma | LDA score (log10) | P |
| Genus |  |  |  | Alloscardovia | 2.0 | 0.04 |
|  |  |  |  | Lachnoanaerobaculum | 2.7 | 0.01 |
|  |  |  |  | Limosilactobacillus | 2.1 | 0.04 |
| Spieces |  |  |  | Campylobacter massiliensis | 2.9 | 0.04 |
|  |  |  |  | Cardiobacterium valvarum | 2.2 | 0.04 |
|  |  |  |  | Capnocytophaga leadbetteri | 3.1 | 0.02 |
|  |  |  |  | Eikenella corrodens | 2.0 | 0.03 |
|  |  |  |  | Gemella morbillorum | 2.1 | 0.04 |
|  |  |  |  | Lachnoanaerobaculum gingivalis | 2.3 | 0.02 |
|  |  |  |  | Selenomonas noxia | 2.5 | 0.04 |
